# Case report: Complete response of a bladder cancer patient with multiple hepatic and pelvic metastases treated by nab-paclitaxel combined with sintilimab

**DOI:** 10.3389/fonc.2022.1020793

**Published:** 2022-12-12

**Authors:** Zhu-lei Tao, Wei Wu, Lin-chun Liang, Jin-feng Pan, Jian-zhou Cao, Xiao-long Jia, Li Fang, Qi Ma

**Affiliations:** ^1^ Medical School, Ningbo University, Ningbo, Zhejiang, China; ^2^ Comprehensive Genitourinary Cancer Center, Ningbo First Hospital, The Affiliated Hospital of Ningbo University, Ningbo, Zhejiang, China; ^3^ Department of Medical Oncology, Mingzhou Hospital, Ningbo, Zhejian, China; ^4^ Department of Urology, Ningbo First Hospital, The Affiliated Hospital of Ningbo University, Ningbo, Zhejiang, China; ^5^ Ningbo Clinical Research Center for Urological Disease, Ningbo, Zhejiang, China; ^6^ Translational Research Laboratory for Urology, the Key Laboratory of Ningbo City, Ningbo, Zhejiang, China; ^7^ Yi-Huan Genitourinary Cancer Group, Ningbo, Zhejiang, China

**Keywords:** case report, nab-paclitaxel, PD-1, metastatic bladder cancer, sintilimab

## Abstract

This article described a patient with metastatic bladder cancer (mBC) who was successfully treated with nab-paclitaxel plus sintilimab. Localized muscle-invasive bladder cancer (MIBC) was discovered in a 56-year-old man who received radical cystectomy and platinum-based adjuvant chemotherapy. Eleven months after cystectomy, this patient developed numerous hepatic and pelvic metastases and progressed to mBC. The patient was given an anti-PD-1 antibody (sintilimab 200mg, q3w) in combination with Nab-paclitaxel (100mg, qw) for mBC. Complete remission (CR) was achieved after nine cycles of therapy, and the patient had no severe side effects during the treatment. The disease remained in CR after 41 months of follow-up. This case suggests that nab-paclitaxel combined with sintilimab is a safe and effective option in treatment of mBC.

## Background

The standard first-line treatment for unresectable locally advanced or metastatic bladder cancer is platinum-based chemotherapy. Due to the toxicity of cisplatin, nearly 50% of patients are unsuitable for cisplatin-based cytotoxic treatment (1). Furthermore, for patients who are treated with platinum-based chemotherapy, if the disease relapse, the median OS for mUC was <9 months (2). If platinum-based first-line chemotherapy fails, the patients are usually suggested to receive second-line therapy. However, second-line chemotherapy such as docetaxel, paclitaxel, nab-paclitaxel, or vinflunine only shows limited effects. The immune checkpoint inhibitors (ICIs) play an emerging role in treatment of mBC. Compared to second-line chemotherapy, programmed cell death 1 (PD-1) / programmed cell death ligand 1 (PD-L1) inhibitors significantly improved the objective response rate (ORR) as second-line therapy for metastatic urothelial cancer (3). Currently, pembrolizumab, nivolumab and avelumab are recommended by European Association of Urology (EAU) and National Comprehensive Cancer Network (NCCN) guidelines for bladder cancer (4, 5).Tislelizumab and toripalimab are also approved in China for second-line treatment of mBC (6, 7). However, the reported objective remission rates (ORR) for single use of ICIs as second-line therapy for mBC are only 17-24% (8).

For first-line treatment of mBC, checkpoint inhibitors atezolizumab and pembrolizumab are recommended to patients who are unfit for cisplatin and are tested PD-L1 positive by NCCN and EAU guidelines (4, 5). NCCN guidelines also recommend atezolizumab and pembrolizumab for patients with locally advanced or metastatic urothelial carcinoma who are not eligible for any platinum-based chemotherapy, regardless of PD-L1 expression. These guidelines also recommend maintenance immunotherapy (avelumab) if non-progressive disease occurs after platinum-based chemotherapy. According to JAVELIN Bladder 100 study (9), comparing to best supportive therapy, adding maintenance avelumab to best supportive care significantly increased overall survival (OS) (median OS, 14.3 months versus 21.4 months).

It is widely believed that some chemotherapy may promote tumor immunity. Chemotherapy increases tumor immunity in two ways: inducing immunogenic cell death or disrupting the strategies that tumors use to evade the immune response (10). ICIs enhance the overall survival (OS) rate of mBC patients considerably, however, the response rate for these drugs remain modest (about 13% to 29%) (11). Many ongoing trials have used PD-1/PD-L1 inhibitors in combination with chemotherapy to find medicines with potential synergistic activity to improve the efficacy of ICIs (12).

We presented a 55-year-old man who underwent radical cystectomy and adjuvant platinum-based chemotherapy after being diagnosed as locally advanced urothelial carcinoma. Only 11 months later, the disease recurred and developed into multiple liver and pelvic metastases. Then the patient was diagnosed as mBC and received Nab-paclitaxel combined with ICIs therapy. After 9 cycles of combined therapy, the patient achieved complete remission (CR) and maintained long-term stability for more than 41 months.

## Case presentation

In January 2018, a 55-year-old male patient came to our department due to recurrent hematuria for six months. The man had been suffering from hypertension for a year, had been smoking and drinking for 30 years, and had no family history of cancer. Enhanced computed tomography urography (CTU) revealed malignant tumor of the bladder and involvement of the lower part of the left ureter. As no metastases were found in this patient, on February 7, 2018, the patient underwent radical cystectomy in a prestigious hospital in Shanghai. Pathology of the specimen showed bladder high-grade invasive urothelial cancer, infiltrating the whole muscle layer of the bladder wall and invading the prostate. Cancer was also detected at the urethral incisal margin. Immunohistochemistry showed PD-L1(SP142) (tumor 50%++, interstitial 5%+), and combined positive score(CPS) of this patient was 5, and the disease was regarded as PD-L1 positive. The patient was eventually diagnosed as bladder high-grade invasive urothelial cancer, and the TNM stage was T4aN0M0. The patient recovered well after surgery, and no postoperative complications occurred. Following the surgery, the patient underwent four cycles of platinum-based adjuvant chemotherapy; the patients had mild fatigue during chemotherapy, and no other adverse events occurred.

**Figure 1:**
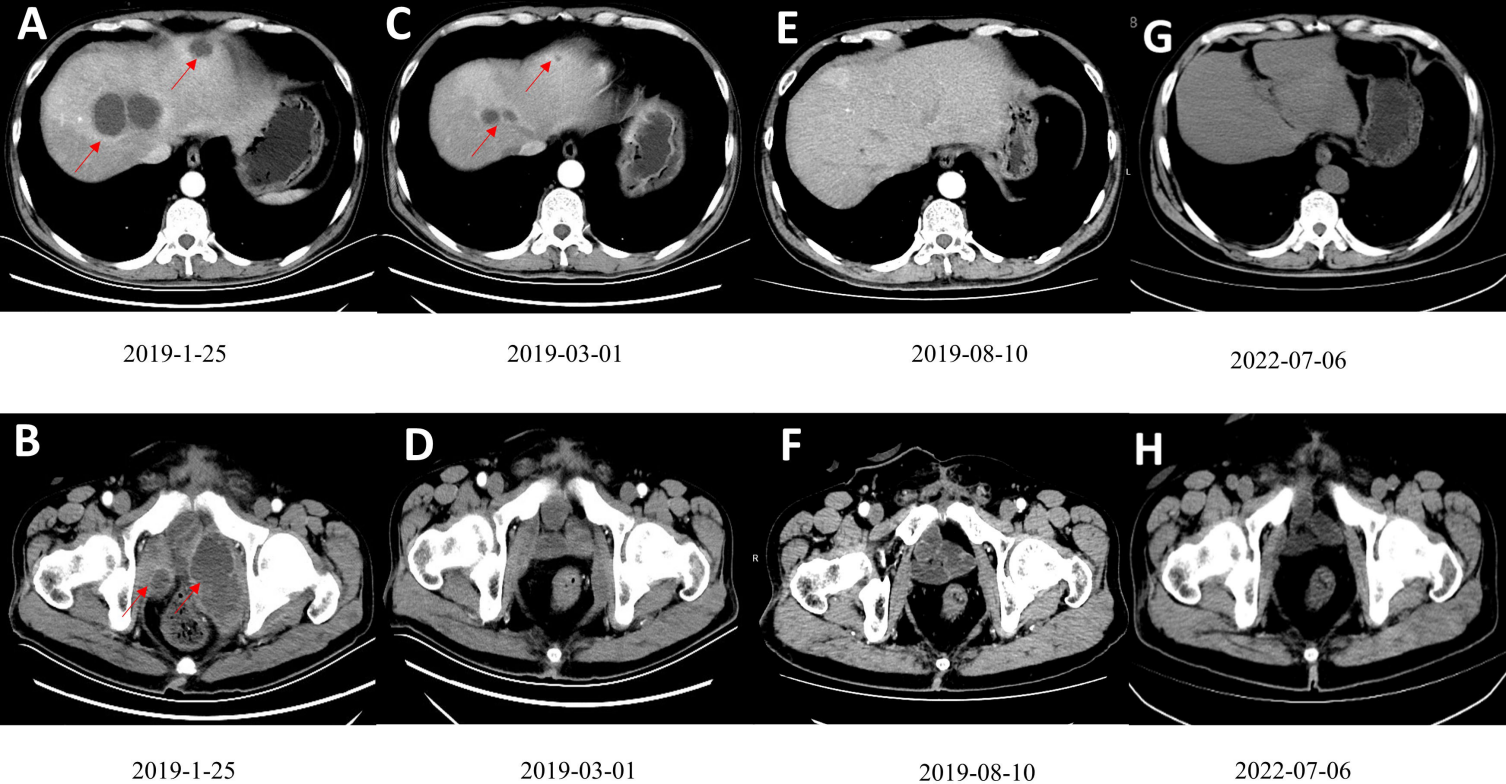
**(A, B)** Contrast-enhanced CT scan showed multiple low-density tumors of various sizes in the pelvis and liver, and the largest liver metastatic tumor was 4.6cm×3.9cm. **(C, D)** A contrast-enhanced CT scan after three courses of treatment showed that the tumors in the pelvis and liver had shrunk significantly. **(E, F)** After ten cycles of treatment, CR was observed. **(G, H).** The patient had been disease free for 41 months. Figures G and F are the most recent follow-up results.

In December 2018, the abdominal enhanced CT and positron emission computed tomography image (PET-CT) showed multiple pelvic metastases. The patient was then transferred to our hospital. In January 2019, the abdomen enhanced CT showed multiple metastases in liver and pelvic cavity (**Figures 1A, B**). After carefully informed and with the patient’s content, the patient started the treatment of anti-PD-1 antibody in combination with Nab-paclitaxel in January 2019. As sintilimab was the only anti-PD-1 antibody available in our hospital at that time, the patient was treated with sintilimab (200mg ivgtt q3w) in combination with Nab-paclitaxel (100mg ivgtt qw) in January 2019. The patient was examined during therapy for routine blood, thyroid, liver, and kidney function, as well as coagulation and serum tumor markers. Following three cycles of treatment, the patient had abdomen CT examination to determine the treatment's efficacy. The evaluation criteria were based on the solid tumor efficacy evaluation criteria (RECIST).

On March 1, 2019, after the patient had finished 3 cycles of combined therapy, the whole abdomen enhanced CT showed that liver and pelvic metastases were reduced significantly (**Figures 1C, D**). On July 21, 2019, nab-paclitaxel was discontinued (8 cycles, 24 chemotherapy doses in total) while sintilimab maintenance monotherapy (200 mg q3w) was continued. On August 10, 2019, after 9 cycles, the patient achieved CR (**Figures 1E, F**). Sintilimab monotherapy (200 mg q3w) was administered for 2 years (32 immunotherapy doses in total) and stopped in January 2021. The patient developed fatigue and mild rash during the treatment, with no serious adverse reactions. The disease has been kept CR for more than 41 months and is still being followed up without recurrence and adverse events (**Figures 1G, H**). The patient’s imaging before and after nab-paclitaxel combined with sintilimab treatment are also presented as **Supplemental Materials (Supplemental Videos 1, 2)**.

## Discussion

The development of ICIs suppressing the interaction between PD-1 and PD-L1 provided new chances for mBC treatment. These drugs demonstrated their role in mBC firstly as second-line therapy. Pembrolizumab was compared to second-line chemotherapy following progression or recurrence of first-line platinum-based chemotherapy in the phase KEYNOTE-045 trial (13). Compared with the chemotherapy group, pembrolizumab group led to a significantly longer OS (median OS, 10.3 months versus 7.4 months). The FDA also approved Nivolumab based on the results of CheckMate 275, with a median PFS of 2 months, a median ORR of 19.6%, and a median OS of 8.74 months. In the subgroup with PD-L1 expression ≥ 1%, the median OS was 11.30 months, and the PD-L1 expression < 1% subgroup had a median OS of 5.95 months. In this study, regardless of the expression status of PD-L1, patients could gain benefits. Even if the tumor expression of PD-L1 was less than 1%, 16.1% of patients showed objective response (14). However, it should be mentioned that the phase III IMvigor 211 trial, which compared atezolizumab to second-line chemotherapy, did not reach its main endpoint and did not improve the OS of patients with high expression of PD-L1 (15). In 2021, FDA announced the withdrawal of second-line atezolizumab indications for metastatic urothelial cancer (16).

After anti-PD-1 monotherapy demonstrated its efficacy as a second-line treatment in mBC, whether PD-1/PD-L1 inhibitors single use is also effective as first-line therapy needs to be further tested. Phase II Keynote-052 evaluated pembrolizumab as first-line therapy in platinum-ineligible patients with mBC, with an ORR of 29% and a CR of 7%. In this study, PD-L1 positivity (using Dako 22C33 test) was defined as CPS ≥10%. Patients with a CPS ≥ 10% had the most obvious response; however, responses were observed in all types of PD-L1 expression (17). The FDA has also approved atezolizumab as a first-line treatment for patients with locally advanced BC/mBC who are not candidates for cisplatin-based chemotherapy, based on the results of the IMvigor 210 single-arm Phase II trial. PD-L1 positivity (using SP142 rabbit antibody) was defined by ≥5% of PD-L1 positive immune cells in tumor microenvironment. Median PFS and OS was 2.7 months and 15.9 months for all patients separately. The objective response rate (ORR) was 23%, while the complete response rate (CRR) was 9%. Atezolizumab showed a long-lasting and tolerable safety profile, but PD-L1 expression was not significantly observed enrichment of response (18).

Although ICIs generated a great optimism in mBC treatment, only a small number of patients have been benefited. There is an urgent need for joint strategies aiming at improvement response rates and biomarkers for predicting responses (19). Increasing evidence supports the use of ICIs in conjunction with chemotherapy in the treatment of cancer. Is this strategy also applicable in mBC? IMvigor130 is a phase 3 randomized trial in which atezolizumab with platinum-based chemotherapy was compared to platinum-based chemotherapy in previously untreated patients with advanced urothelial cancer (20). KEYNOTE-361 is another phase 3 randomized trial which also used to evaluate the efficacy of Pembrolizumab with platinum-based chemotherapy comparing to platinum-based chemotherapy in patients with advanced urothelial carcinoma who have previously untreated disease (21). Unfortunately, both studies showed disappointing results. However, in the JAVELIN Bladder 100 study, maintenance avelumab treatment significantly prolonged overall survival of the patient after platinum-based first-line chemotherapy (11). Thus, chemotherapy followed by sequential immunotherapy, rather than combined with immunotherapy, was recommend for first-line treatment of mBC.

Recent investigations have demonstrated that the antitumor efficacy of combining anti-PD1 antibodies and Nab-paclitaxel worth further investigation. For patients with stage IV squamous cell lung cancer, KEYNOTE-407 study showed that pembrolizumab plus chemotherapy (carboplatin and paclitaxel or nab-paclitaxel) significantly improved OS and PFS (median OS, 15.9 months vs 11.3 months; median PFS, 6.4 months vs 4.8 months), regardless of the level of PD-L1 expression (22). Impassion130 evaluated atezolizumab + nab-paclitaxel as first-line treatment for patients with unresectable locally advanced or metastatic triple-negative breast cancer. Compared with placebo plus nab-paclitaxel, atezolizumab + nab- paclitaxel improved OS and PFS (median OS, 21.3 months vs 17.6 months; median PFS, 7.2months vs 5.5 months) (23). The above studies show that nab-paclitaxel combined with anti-PD1/PD-L1 antibodies can prolong the survival time of patient.

Nab-paclitaxel and PD1/PDL1 inhibitors may also be a choice for mBC. The PEANUT study evaluated pembrolizumab in combination with nab-paclitaxel as second-line therapy for mBC. The study demonstrated excellent safety, durable PFS (5.9 months), and clinically meaningful ORR (38.6%). Tumor mutation burden and CPS are not significantly associated with PFS at univariable analyses (24). The response was more pronounced in patients with CPS ≥ 10% than in patients with CPS < 10% (77.8% versus 30.4%). These results suggested that Nab-paclitaxel chemotherapy combined with ICIs improved clinical efficacy as second-line treatment after platinum chemotherapy failure, regardless of the expression status of PD-L1.

In this case, though the patients received platinum-based chemotherapy, the disease progressed to mBC quickly. Although sintilimab has not been approved for metastatic urothelial carcinoma in China, it has shown reliable efficacy and safety in clinical trials in solid tumors. Under the medical conditions at that time, sintilimab was the only anti-PD1 antibody available in our hospital. The patient received nab-paclitaxel and sintilimab based on our previous experience (25). The patient was well informed and was aware of these medications' off-label use. With the patient’s written consent, we treated the patient with Nab-paclitaxel in combination with sintilimab, and the results exceeded our expectations. The patient achieved CR and remained disease free for over 41 months.

In conclusion, this case demonstrated that Nab-paclitaxel, in combination with sintilimab, may be a safe and effective treatment option for mBC patients. More clinical data are needed to support this treatment strategy.

## Data availability statement

The original contributions presented in the study are included in the article/**Supplementary Material**. Further inquiries can be directed to the corresponding authors.

## Ethics statement

The studies involving human participants were reviewed and approved by the ethical review committee of Ningbo First Hospital (No. 2022RS115).

## Author contributions

Conception/design: QM. Provision of study materials or patients: QM, WW, and L-CL. Collection and/or assembly of data: QM, J-ZC, JFP, WW. Data analysis and interpretation: ZLT, LF and QM. Manuscript writing: ZLT, X-LJ, QM. Final approval of manuscript: All authors. All authors contributed to the article and approved the submitted version.
